# Agricultural Food Production Diversity and Dietary Diversity among Female Small Holder Farmers in a Region of the Ecuadorian Andes Experiencing Nutrition Transition

**DOI:** 10.3390/nu12082454

**Published:** 2020-08-15

**Authors:** Christopher L Melby, Fadya Orozco, Jenni Averett, Fabián Muñoz, Maria José Romero, Amparito Barahona

**Affiliations:** 1 Department of Food Science and Human Nutrition, Colorado State University, Fort Collins, CO 80523, USA; 2 Colorado School of Public Health, MPH Program at Colorado State University, Fort Collins, CO 80523, USA; jenniaverett@gmail.com; 3 School of Public Health, Universidad San Francisco de Quito, Quito, Ecuador; forozco@usfq.edu.ec; 4 Visor Análisis Estadístico, Quito, Ecuador; fabian_munoz@yahoo.com; 5 Faculty of Engineering in Agricultural and Environmental Sciences, Universidad Técnica del Norte, Ibarra, Ecuador; mjromero@utn.edu.ec; 6 Faculty of Health Sciences, Universidad Técnica del Norte, Ibarra, Ecuador; amparibmj@hotmail.com

**Keywords:** nutrition, food crop diversity, dietary diversity, food insecurity, women, Ecuador

## Abstract

Some rural areas of Ecuador, including the Imbabura Province of the Andes Highlands, are experiencing a double burden of malnutrition where micronutrient deficiencies persist at the same time obesity is increasing as many traditional home-grown foods are being replaced with more commercially prepared convenience foods. Thus, the relationships among agricultural food production diversity (FPD), dietary diversity (DD), and household food insecurity (HFI) of the rural small holder farmers need further study. Therefore, we examined these associations in small holder farmers residing in this Province in the Andes Highlands (elevation > 2500 m). Non-pregnant maternal home managers (*n* = 558, x age = 44.1, SD = 16.5 y) were interviewed regarding the number of different agricultural food crops cultivated and domestic animals raised in their family farm plots. DD was determined using the Minimum Dietary Diversity for Women Score (MDD-W) based on the number of 10 different food groups consumed, and household food insecurity (HFI) was determined using the 8-item Household Food Insecurity Experience Scale. The women reported consuming an average of 53% of their total food from what they cultivated or raised. Women with higher DD [MMD-W score ≥ 5 food groups (79% of total sample)] were on farms that cultivated a greater variety of crops (x = 8.7 vs. 6.7), raised more animals (x = 17.9 vs. 12.7, *p* < 0.05), and reported lower HFI and significantly higher intakes of energy, protein, iron, zinc, and vitamin A (all *p* < 0.05). Multiple regression analyses demonstrated that FPD was only modestly related to DD, which together with years of education, per capita family income, and HFI accounted for 26% of DD variance. In rural areas of the Imbabura Province, small holder farmers still rely heavily on consumption of self-cultivated foods, but greater diversity of crops grown in family farm plots is only weakly associated with greater DD and lower HFI among the female caretakers.

## 1. Introduction

To ensure adequate nutrient intake conducive to health, eating patterns that include a diversity of food groups is considered important [1]. On the other hand, lower dietary diversity increases the probability of inadequate intake of nutrients, especially protein, vitamins, and minerals. Globally, nearly two billion people suffer from micronutrient deficiencies which contribute to a large societal health burden resulting in impaired cognitive and physical development, weakened immunity in children [2] and adults, lower school performance, and lost productivity [3]. Maternal undernutrition contributes to fetal growth restriction and the totality of maternal and childhood undernutrition (energy and micronutrient deficiencies, stunting, wasting, and inadequate breast feeding) in low and middle-income countries contributes to millions of deaths each year [4]. Additionally, both maternal and childhood obesity are increasing, which coupled with micronutrient and protein deficiency increase risk for both communicable and non-communicable diseases [4].

Ecuador, like many other countries, is experiencing a double burden of malnutrition [5,6]. Micronutrient deficiencies persist at alarming rates at the same time the nutrition transition away from ancestral dietary patterns to one characterized by greater consumption of foods high in sugar and fat is driving increasing rates of overweight, obesity, and non-communicable diseases [6,7,8]. More than one-third of overweight women exhibit micronutrient deficiencies, 12.6% of overweight or obese mothers have a child with anemia and 14% have a child with zinc deficiency [7]. For children less than 5 years, the prevalence of stunting, anemia and zinc deficiency are higher in rural areas, among Indigenous children, and in the poorest quintile of the population [6]. According to results from Ecuador’s National Health and Nutrition Survey (ENSANUT-ECU) food consumption patterns in the population are contributing to the double burden [6]. Of note is an excessive intake of refined carbohydrates, mainly white rice and white bread, along with an inadequate intake of fruits and vegetables [6,7]. Thus, a limited diversity of dietary staples provides a large percentage of low nutrient-density calories [6,9]. Protein, iron, zinc, and vitamin A are the nutrient deficiencies most common in this population [6]. Additional factors contributing to poor nutrition include poverty, food insecurity, and a lack of dietary diversity in the resource-poor subset of the population [10,11]. Not surprisingly, food insecurity in urban neighborhoods in Ecuador was recently shown to be associated with under nutrition and low dietary diversity [12]. Indigenous groups in Ecuador are disproportionately affected by poverty and food insecurity. An estimated 60% of Indigenous families are below the national poverty threshold compared to a national rate of 30% [6]. Many are smallholder farmers who rely on their own agricultural production as a primary source of food and food security, although this phenomenon is changing with the nutrition transition fueled by increasing availability of more commercially prepared foods, changes in cultural preferences and practices, less knowledge regarding preparation of traditional ancestral food, greater consumption of highly palatable processed food as a matter of status, migration from the countryside to the city, and greater ease of transportation to urban areas [13,14].

Two recent reviews of studies conducted in countries primarily located in Africa, but in some countries in Asia and Latin America [13,14], have examined the relation between agricultural diversity and dietary diversity, with some positive associations found in some cases but not others. Possible reasons for inconsistent findings include a variety of external factors such as climate, altitude, soil quality, farming methods, proximity to food markets, market orientation (e.g., market favorable for cash crops), among others, and also those factors related to the farmers themselves such as socio-economic status, transportation availability, farming and food preparation skills, and degree of adherence to more traditional family food consumption patterns versus access to commercially-prepared foods. Jones et al. [15] reported a weak positive association between farm-level agricultural diversity and dietary diversity scores of women in the Peruvian Andes, but little work on this issue has been done in Ecuador. In 2015, we conducted a study [8] examining rural versus urban food consumption patterns in the Chimborazo Province, which has one of the highest rates of poverty in Ecuador, low education levels, and limited access to transportation from rural areas to urban markets. Sixty-five percent of the rural-dwelling women reported obtaining the majority of their food from self-cultivation, while only 8% of the urban dwellers obtained the majority of their food from what they grew themselves [8]. Not surprisingly, Oyarzun et al. [16] found, “a positive correlation between number of on-farm species and family-level dietary diversity” in the rural areas of this same province. Nevertheless, convenience food shops, even in remote rural areas have been found to offer low nutrient density foods high in fat, sugar, and salt; thus reliance on self-cultivated food crops may be decreasing [8].

It is unclear if agricultural food production diversity of small holder farms is related to DD in rural areas of Ecuador where poverty is all too common, but non-agricultural commerce (craftsmanship and small business enterprise) has become an important driver of the local economy and the nutrition transition appears to be accelerating. One such area is the Imbabura Province of the Central Highlands of Ecuador. This area, in contrast to the Chimborazo Province, is characterized by greater urban expansion into agricultural areas, greater small business commerce, greater access to food markets and to commercially prepared foods and higher prevalence of overweight and obesity [6]. The construction industry and commerce linked to handicrafts (textiles, jewelry, leather, woodworking, and artwork) and tourism are important to the economy of the region, with the agricultural sector representing a smaller contribution to economic activity [17]. Also, 49% of the total agricultural production units in Imbabura have an area of less than 1 hectare (1000 square meters) [18], which could limit the quantity and diversity of agricultural food production, possibly resulting in lower intakes of self-cultivated foods, even when household food insecurity (HFI) may be present. It is unclear if small holder farmers in this region obtain less food from self-cultivation in favor of more commercially processed foods, a phenomenon characteristic of the nutrition transition, which could obviate a link between FPD and DD and contribute to overweight and obesity. Therefore, the objectives of this study were to (1) determine the magnitude of agricultural food production diversity (FPD); (2) determine the extent to which female home managers of small holder farms rely on self-cultivated foods; and (3) examine the relationships among DD, agricultural FPD, socioeconomic level, and household food insecurity (HFI) in women living in rural areas above 2500 m in the Imbabura Province in the Andes Highlands of Ecuador. Gaining a better understanding of these relationships, the agricultural practices, and the primary sources of food consumed can help guide development of agricultural and public health initiatives and policies that seek to alleviate the burden of under and over nutrition within this population.

## 2. Materials and Methods

### 2.1. Study Design and Participants

**Figure 1:**
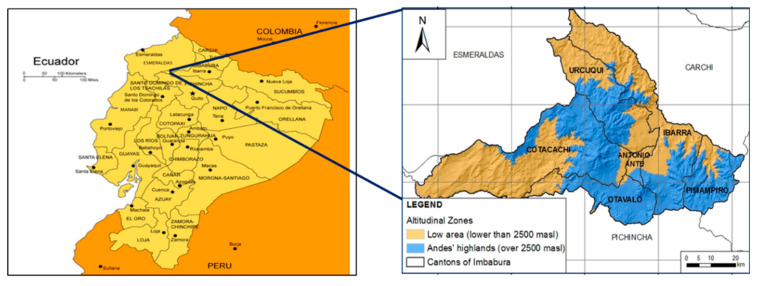
Map of the areas in the Imbabura Province of Ecuador >2500 m above sea level (blue regions) from which the sample of small holder farmers was obtained. (www.freeusandworldmaps.com).

This cross-sectional study was conducted in 12 parroquias (political subdivisions) located in six cantons (Figure 1) situated above 2500 m in the Imbabura Province of the Ecuadorian Andes Highlands, primarily populated by Indigenous Ecuadorians. These parroquias were chosen in order to obtain a representative sample of areas in the Province where small holder farming is often more difficult due to the elevation and steep slopes of the cultivated hillsides. A previous unpublished pilot study was conducted in this region March through May of 2015 for purposes of identifying the magnitude of agricultural diversity on small holder farms. For this pilot study, presidents of each of the 12 parroquias provided a list of small holder farm households and from the respective lists, households were randomly sampled using GIS mapping. Agricultural crop diversity ranged from cultivation of only 2–3 different species to up to 40 different species on individual farms. For the current study, permission was again obtained from the respective parroquia presidents, and the same households were again selected and surveyed from March to May 2018, using the same GIS mapping previously used by a member of the research team (MJ Romero). When the potential maternal study participant from the identified household was not available, the female caretaker in the nearest household located to the right was interviewed. Highly trained agronomy and nutrition professionals were used as research assistants to collect data. They were local to the area and understood the common agricultural practices and possessed key cultural and nutrition-related knowledge. Several of the assistants spoke Kichwa, the primary Indigenous language of this area and were especially helpful in communicating with some of the older women who were more comfortable communicating in Kichwa than Spanish. The assistants explained the purpose of the study and requirements for participation, read the consent form to women, answered any questions, and for those willing to participate, obtained written informed consent. For those who were unable to write, a thumb print in lieu of a signature was collected. Research was approved by the Institutional Review Board and Bioethics Committees at both Colorado State University (CSU protocol #17-7492H) and the Universidad San Francisco de Quito (USFQ protocol #2017-142IN), respectively. The study sample consisted of a total of 558 non-pregnant, maternal caretakers between the ages of 18–85 years with a mean age of 44 years (SD = 16.3). Mean body mass index was 27.1 kg/m^2^ (SD = 4.3). Forty-four percent of the women were classified as overweight (BMI: 25–29.9 kg/m^2^) and 23% classified as obese (BMI > 30 kg/m^2^). The study was delimited to women due to their important role in food acquisition and preparation for their families and because many men in the selected rural areas are often employed away from home and not available for study participation. Eighty-four percent of the women reported their daily work was household management and/or farming. Seventy-two percent of the women self-identified their ethnicity as Indigenous, 27% identified as Mestiza, and 1% as other. There were no notable differences in demographic, anthropometric, dietary, and agricultural farm characteristics among ethnic groups, thus all ethnic groups were examined together as a single sample.

### 2.2. Specific Procedures

Five research teams were trained for data collection by the study investigators from the Universidad San Francisco de Quito (USFQ), the Universidad Técnica del Norte (UTN), and Colorado State University (CSU). Each research team consisted of two members, one nutritionist and one agronomist, who underwent extensive training and practice regarding appropriate data collection procedures.

#### 2.2.1. Survey Instrument

The survey instrument was developed to record sociodemographic variables, anthropometric measures, dietary and agricultural data, and perceptions of household food insecurity. It was patterned after a survey instrument used in a previous study examining characteristics of the nutrition transition in urban and rural areas of the Chimborazo region of the Ecuadorian Andes Highlands [8].

#### 2.2.2. Dietary Intake

Dietary intake was determined using a multiple pass 24-h dietary recall. Participants were interviewed by a nutritionist and asked to report all foods and beverages consumed the previous day. The interviewers were nutrition professionals, educated at the Universidad Técnica del Norte (Technical University of the North), which is the major comprehensive university in the Province. The nutritionists were familiar with the dietary patterns in the province and used various measuring utensils, plates, cups, and bowls.to help respondents report as accurately as possible the different types and amounts of foods and beverages consumed during the previous day. The nutritionists were instructed to probe for information regarding serving sizes, second helpings, methods of food preparation including boiling, frying, and roasting, types and amounts of oil used in cooking, snacks consumed, individual ingredients used in casserole type dishes and soups, and unsweetened and sugar-sweetened beverage consumption included aromatic water (agua aromatica, herb-infused water usually sweetened with sucrose). Dietary data recorded from all 24-h recalls were entered into a database for nutrient analysis (total kcalories, g of macronutrients, mg of iron and zinc, and retinol equivalents of vitamin A) using food values developed in the Encuesta Nacional de Salud y Nutrición en Ecuador (ENSANUT-ECU 2012) [6].

#### 2.2.3. Dietary Diversity and Food Insecurity

The magnitude of dietary diversity (DD) was calculated from the 24-h dietary recalls using the Minimum Dietary Diversity for Women (MDD-W) indicator [10], which is based on consumption of foods from 10 different food groups (examples of commonly consumed foods in parentheses): (1) Grains, white roots and tubers, and plantains (white bread, potatoes, rice, maize, quinoa, white sweet potato) (2) Pulses (legumes, lupini beans) (3) Nuts and seeds (peanuts and tree nuts), (4) (chicken and quail), Dairy (milk, yogurt, cheese), (5) Meat, poultry and fish (beef, chicken, pork, guinea pig), (6) Eggs, (7) Dark green leafy vegetables (chard, spinach, broccoli), (8) Other vitamin-A rich fruits and vegetables (pumpkin, orange sweet potato, papaya, tree tomatoes, carrots), (9) Other vegetables (cabbage, onion, cucumber), and (10) Other fruits (apple, banana, pineapple). Using this approach, a food is counted in its respective food group if ≥15 g of the food was consumed. Possible dietary diversity scores (DDS) range from 0–10, with a score of 5 or greater deemed to be the minimum associated with adequate micronutrient intake among women of child-bearing age. Household food insecurity (HFI) was assessed using the 8-item Spanish Household Global Food Insecurity Experience Scale (FIES) developed by FAO [19,20]. Affirmative responses to each of the 8 questions indicate greater food insecurity, with a range of possible scores from 0–8 going from lowest to highest levels of food insecurity.

#### 2.2.4. Agricultural Diversity on Small Holder Farms

The diversity of crops grown on the farms was determined by both interview and actual observation of crops by the agronomist research assistants when visiting each farm. Each study participant was asked to identify all food crops, fruit trees, and plants used for alimentation currently being grown on their farms. These crops were recorded and additionally the agronomist further probed about plant foods commonly grown in this region and recorded any additional crops, trees, plants they observed to be growing on the smallholder farms, both in larger plots and in smaller domestic gardens, that were not identified in the interview. Respondents were also asked to identify the types and number of animals on their farms raised for food. They were specifically asked whether or not they raised any of 10 different species of animals (chickens, guinea pigs, rabbits, ducks, turkeys, quail, cattle, sheep, goats, and pigs), from which food could be obtained including animal flesh, internal organs, eggs, and dairy products (cow’s and goat’s milk). Three specific variables were calculated to describe food production diversity of the farms: (1) the number of *different* crops grown for food; (2) the total number of animals raised for food; and (3) the food production diversity (FPD) score, which was determined by categorizing the cultivated crops and animal food products (animal flesh and organs, dairy, and eggs) on each small holder farm according to the same 10 food groups used to determine DDS [21]. Possible FPD scores ranged from 0–10 going from lowest to highest production diversity.

#### 2.2.5. Physical and Demographic Characteristics

To describe the physical characteristics of the participants, height, weight, and body mass index (BMI) were determined. Body weight was measured using a portable digital scale to the nearest 0.1 kg with participants wearing light clothing. Participants were asked to remove shoes, hats, jackets, sweaters, and jewelry. It was not possible to obtain nude weights of the participants and because traditional lightweight clothing among rural Ecuadorian women includes multiple layers estimated to weigh approximately 1.0 kg, a kg was subtracted from the measured weight of each participant. While certainly not exact, this approach to quantifying weight more accurately represents the participants’ true body weights. Height was measured to the nearest 0.1 cm using a portable stadiometer with participants standing erect with the head position in a Frankfort horizontal plane. BMI was calculated as weight (kg)/height (m)^2^. Respondents were asked to estimate their usual monthly family monetary income from all possible sources. This value was then divided by the total number of family members living in the home to determine the per capita family monetary income. Respondents also reported the number of years of formal education they completed, which ranged from 0–18 years.

### 2.3. Statistical Analyses

Data were numerically coded, entered in a spreadsheet, and checked for errors and outliers. The data were then transferred into an IBM SPSS (IBM Corporation, version 26, Armonk, NY, USA) spreadsheet and analyzed. Multivariate Analysis of Variance (MANOVA) was used to examine differences in the characteristics of the women and the agricultural practices between those who met the minimum dietary score for women [Higher Dietary Diversity (HDD) = MDD−W score ≥ 5, i.e., consumption of ≥5 of the 10 possible different food groups] and the women who did not meet the minimum [Lower Dietary Diversity (LDD) = MDD−W score < 5, i.e., consumption of <5 of the 10 possible different food groups]. Chi-Square analyses were used to examine the percentages of LDD and HDD women both producing and consuming different food groups. Simple correlation analyses were used to examine the strength of the associations among DD (number of food crops cultivated, total number of farm animals raised for food or food products, agricultural FPD score, HFI, age, education, and per capita income. Those variables significantly correlated with DD were used in a stepwise multiple regression analyses to determine the significant multivariate predictors of DD. The data used in the multiple regression model were found to meet the necessary assumptions to ensure robustness of the model. There were as many as 6 missing values, depending on the variable examined. Where appropriate, data are presented as means and standard deviations (SD). Statistical significance was set at *p* < 0.05.

## 3. Results

Participants estimated the percentage of their food intake obtained from 6 different venues. They reported consuming an average of 53% (SD = 19%) of their food from among those plant crops cultivated and animals raised on their small holder farms, 33% (SD = 18%) from food markets, 11% (SD = 11%) from small neighborhood general stores, 2% (SD = 5%) from trading with neighbors, 1% (SD = 5%) from large supermarkets, and less than 1% (SD = 5%) from commercial restaurants. The FPD score for the small holder farms was positively but weakly correlated with percent of food consumed from self-production (r = 0.20, *p* < 0.001) and negatively correlated with the percent of food obtained from markets (r= −0.19, *p* < 0.001). However, there was no correlation between DD and percent of food consumed from self-cultivation or from markets.

Table 1 shows the mean values for the demographic and physical characteristics of all study participants and also the same women divided into two groups based on their DD scores determined from the food groups identified from their 24-h dietary recalls (Lower Dietary Diversity: LDD = MDD−W score < 5 food groups, *n* = 114, 20% of entire sample); and Higher Dietary Diversity: HDD = MDD−W score > 5 food groups, *n* = 438). The LDD group consumed an average of only 3.4 different food groups during the 24-h dietary-recall period compared to 6.3 food groups for the HDD women. The LDD women were significantly older, had less years of formal education, exhibited lower body weight and ingested lower intakes of kcalories, protein, iron, zinc, and vitamin A. Poverty was present in both groups with a mean per capita monetary income significantly lower for families of LDD women (50 US$/month, SD = $34) compared to HDD (72 US$/month, SD = $76).

**Figure 2:**
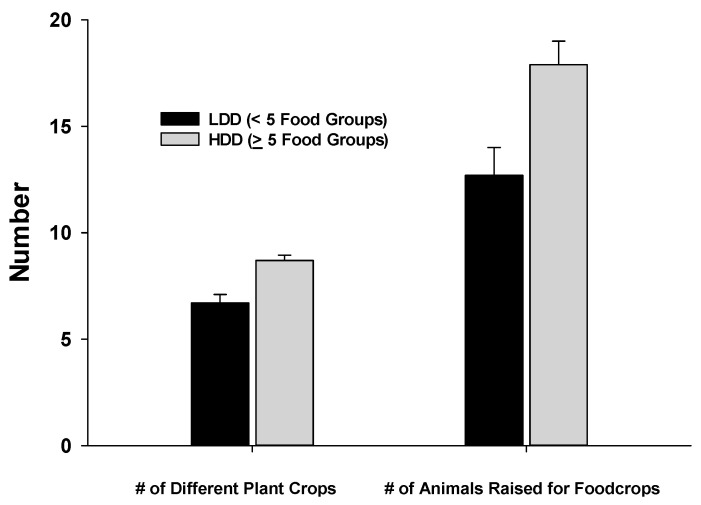
Number of different types of plant food crops cultivated and total number of animals raised by women who exhibited low dietary diversity (LDD: <5 food groups) compared to women with higher dietary diversity (HDD > 5 food groups consumed). Differences were significant at *p* < 0.05.

There was variation in the magnitude of the FPD score among the small holder farms (x = 4.68 out of a possible 10 food groups, median = 5 food groups), but the overall diversity based on this calculated value was low. However, there was greater diversity in the total number of different crops cultivated, ranging from a single crop to 40 different types of crops cultivated (Median = 8 different crops). There was larger variability in the total number of animals raised for food products, ranging from 0–200 (one farm raised 200 chickens for eggs). Figure 2 shows that the farms of HDD women cultivated a significantly greater number of crop varieties and raised a greater number of animals for food than farms of LDD women. The FPD score was also significantly higher for the HDD women [x = 4.8 food groups (SD = 1.7)] compared to the LDD group [x = 4.2 food groups (SD = 1.4, *p* < 0.01)].

**Figure 3:**
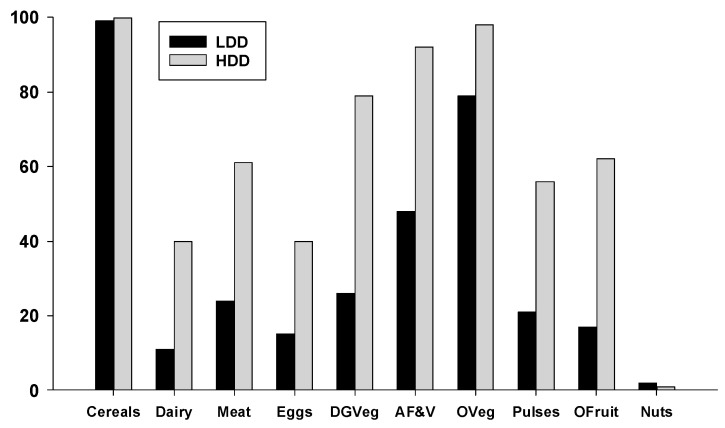
Percentage of LDD and HDD women home managers from small holder farms who consumed each of the 10 different food groups used to determine dietary diversity. All group differences are significant (Chi-square analyses, *p* < 0.0001) except for cereals, grains and nuts/seeds. Abbreviations: Cereals = grains, white roots and tubers and plantains; Meat = meat, fish, poultry; DGVeg—dark green vegetables; AF&V = vitamin A-rich fruits and vegetables; OVeg = other vegetables; OFruit = other fruits; Nuts = nuts and seeds.

To characterize the differences in food consumption within the LDD and HDD groups of women we examined the percentage who consumed each of the 10 possible food groups used to calculate their dietary diversity scores. As shown in Figure 3, almost all the women in both groups consumed foods from the cereal/white roots/tubers/plantain group (LDD = 99%, HDD = 100%), primarily in the form of white rice, corn, potatoes and white bread. Significantly higher percentages of HDD women consumed all other food groups relative to the LDD group (*p* < 0.05), except for nuts and seeds, which were consumed by less than 2% of women. Particularly striking were the low percentages of LDD women consuming dairy (11%), meat, fish, or poultry (24%), eggs (15%), dark green vegetables (26%), vitamin-A rich fruits and vegetables (48%), pulses (21%), and other fruits (17%).

**Figure 4:**
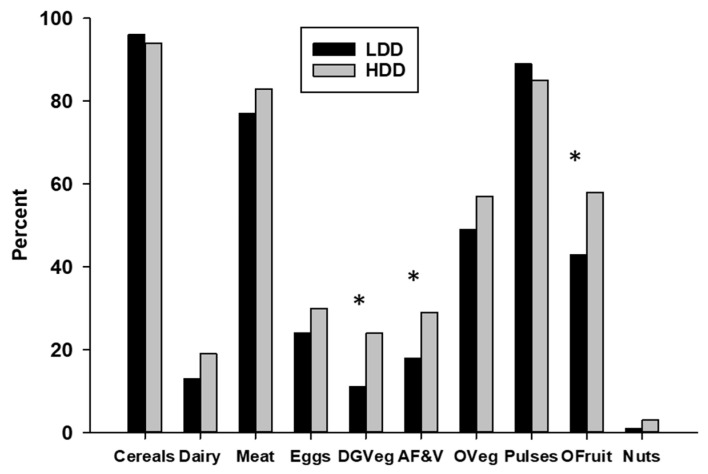
Percentage of LDD and HDD female small holder farmers who produced each of the 10 different food groups used to determine food crop diversity. * Chi-Square analyses, *p* < 0.05) LDD vs. HDD. Abbreviations: Cereals = grains, white roots and tubers, and plantains; Meat = meat and poultry; DGVeg = dark green vegetables; AF&V = vitamin A-rich fruits and vegetables; OVeg = other vegetables; OFruit = other fruits; Nuts = nuts and seeds.

To further characterize agricultural food production diversity, we determined the percentage of small holder farms producing each of the 10 food groups used to determine the FPD score (Figure 4). 

The grains and tubers group (cultivated on 95% of farms), pulses group (cultivated on 86% of farms), meat/fish/poultry group (raised on 82% of farms), and other fruits and other vegetables groups (both grown on 55% of farms) were the most commonly produced food groups. Twenty-eight percent of the farms produced eggs, but the dairy products, nuts and seeds, and dark green vegetable groups were produced by less than 25% of the small holder farms. All food groups except the cereals and tubers and the pulses were produced by a higher percentage of HDD compared to LDD women, although the differences only reached statistical significance for three of the food groups: dark green vegetables (HDD = 24%, LDD = 10%, Chi-Square = 9.8, df = 1, *p* = 0.002), vitamin A-rich fruits and vegetables (HDD = 29%, LDD = 18%, Chi-Square = 4.8, df = 1, *p* = 0.03), and other fruits group (HDD = 58%, LDD = 43%, Chi-Square = 7.8, df = 1, *p* = 0.005).

We calculated the percentage of women who ingested each of the food groups produced on their respective farms. Ninety-five percent of the women whose farms cultivated crops in the cereal grains, white roots, and tubers group consumed food from this group. The percent of women consuming the other food groups from their own cultivated groups were: 95% for other vegetables, 85% for vitamin A-rich fruits and vegetables, 75% for dark green vegetables, 61% for other fruits, 50% for meat, fish, and poultry, 49% for pulses, 43% for dairy, and 34% for eggs. Of the 15 farmers who produced nuts and seeds, only one reported consumption of this food group.

Regarding animal husbandry, 85% of the small holder farms raised at least one of the 10 possible animals for food, with chickens raised on 72% of farms, guinea pigs on 52%, pigs on 45%, cows on 36%, sheep on 10%, and rabbits on 5% of the farms. Ducks, turkeys, and quail were raised by less than 2% of the farmers. There were no differences (*p* > 0.05) between the LDD and HDD groups regarding the mean number of any of the species of animals raised on their respective farms, primarily owing to the greater variability in number of animals on the HDD farms. The mean number of animals raised and the ranges provided for the most commonly raised animals were: chickens (LDD: x = 5.8, range = 0–30; HDD: x = 8.2, range = 0–200), guinea pigs (LDD: x = 4.9, range = 0–50; HDD: x = 6.7, range = 0–100), pigs (LDD: x = 0.7, range = 0–12, HDD: x = 1.1, range = 0–20), and cows (LDD: x = 0.75, range = 0–9, HDD: x = 1.15, range = 0–20).

Simple correlation analyses were performed to identify variables significantly associated with the DD score of the women based on their consumption of 10 possible food groups. Energy intake (r = 0.402, *p* < 0.001), years of formal education (r = 0.301, *p* < 0.001), per capita family income (r = 0.204, *p* < 0.001), total number of different agricultural plant crops cultivated (r = 0.255, *p* < 0.001), total number of animals raised (r = 0.092, *p* < 0.04), FPD score (r = 0.19, *p* < 0.001), age of the women (r = −0.246, *p* < 0.001), and household food insecurity (r = −0.239, *p* < 0.001) were all significantly correlated with DD, although the correlation coefficients are quite small, individually explaining little variability in DD. Years of education, per capita family income, and HFI were not correlated with the FPD score, but HFI was negatively correlated with both education (r = −0.23, *p* < 0.001) and per capita family income (r = −0.25. *p* < 0.001).

Because there are many factors that can influence food intake, we used stepwise (forward) multiple regression analysis to determine if any of the measures of agricultural diversity were associated with DD scores independent of other variables and to determine the total percent of the variance in DD scores explained by multiple variables in combination. (Table 2 and Table 3). All the variables that were correlated with the DD score from the simple correlation analyses were initially entered in the regression model. Total energy intake contributed to the greatest amount of variance explained in the model, followed by HFI, years of education, number of different crops cultivated, and per capita family monetary income. Thus, while the number of crops cultivated as one measure of agricultural diversity was related to the DD score, its contribution to explaining the DD score variability was modest. There was significant shared variability among the five significant independent contributors to the model, with all five together explaining 26% of the variance. The total number of agricultural plant crops cultivated was a better predictor of the DD score than was the FPD score. Also, the number of animals raised for food did not contribute to explaining the variance in the DD score in the multiple regression model.

## 4. Discussion

We chose to study rural-dwelling women residing on small holder farms in the Imbabura province of the Ecuadorian Highlands because little is known about the relation between agricultural production diversity, dietary diversity, and household food insecurity in an area of Ecuador where small holder farm plots are shrinking, farms are located at high altitude, some small holder farming has given way to craftsmanship and entrepreneurship linked to tourism, and the nutrition transition away from ancestral traditional foods to more commercially prepared foods is rapidly occurring. Thus, we considered the possibility that female household managers on these small holder farms might obtain less food from self-cultivation in favor of more commercially processed foods, which could weaken the relation between agricultural FPD and DD, and also contribute to overweight and obesity.

The major findings of our study are: (1) There is considerable variation in the magnitude of agricultural diversity between small holder farms with some farmers growing only a few crops and raising few animals, while others cultivate a wide variety of plant foods and raise a large number of animals for food products. (2) On average, the female household managers reported obtaining about half of their food from the crops they grow and the animals they raise, with the other food coming primarily from urban markets and convenience stores located in their rural areas. (3) Women exhibiting HDD (79% of total sample) cultivated a greater number of different food crops, raised more animals for food production, and exhibited a HFPD score. They also reported less food insecurity and higher intakes of protein and several key micronutrients. These data, along with the positive association of DD with number of food crops cultivated found in the regression analysis, suggest that DD of the female farm managers and agricultural diversity of their farms are positively related. However, this is not to say that crop diversity of small holder farms is a major determinant of DD among female farmers in this region. Indeed, the variability in DD of these women explained by the number of food crops grown on the small holder farms was quite small.

It may not be surprising that among small holder farmers who reportedly depend on an average of half of their food coming from what they produce, agricultural food production diversity is not more strongly related to dietary diversity. The relatively low food production diversity score (x = 4.68 out of a possible 10; Median = 5) is a possible reason, as lack of adequate heterogeneity (e.g., multiple crops grown on the farm from the same food group, such as corn and potatoes) would preclude finding a strong relationship. These farms were all situated at high altitude, which could limit the diversity of crops grown. Increasingly in Imbabura, there is greater accessibility of public and private transportation from rural areas to urban food markets, and also increased availability of small food markets and convenience stores in rural areas. Together, they afford purchasing and trading of food stuffs between farmers, a phenomenon that could enhance dietary diversity among those whose farms exhibit little food crop variety. Indeed, Oyarzun et al. {16] found a high conversion of farm production into cash in order to purchase foods at various markets and grocery stores. Greater access to commercially prepared foods could also limit dietary diversity and quality if less nutritious foods like confectioneries, chips, oils, noodles, and sugar-rich beverages are purchased with monies earned from selling healthier cash crops, including meat and poultry, fruits, vegetables, whole grains, and legumes. In a previous study, we found the shift from traditional ancestral food patterns to more convenience food choices to be extending into rural areas of Ecuador as well [8]. Despite widespread availability of fruits and vegetables in Ecuador, consumption of these nutrient-rich foods is low in many areas of the country [6,7]. Ochoa-Aviles et al. [22] have shown that many highly processed food items, because of increased availability in local markets and their high palatability, are “crowding out” more nutritious foods from the diets of Ecuadorian adolescents, a phenomenon that also appears to be occurring in Ecuadorian adults [6,7]. These food items including sugar-sweetened beverages, pastries, crackers, chips, and candy are now produced in Ecuador by national and multi-national food companies. This phenomenon is similar to that reported by Webb et al. [23] of significant market penetration of highly processed, pre-packaged snack foods in rural Guatemala, which were reported as displacing more nutritious foods.

In the present study, we also found that consumption of self-cultivated food was dependent on the *types* of food produced. Virtually all the women consumed foods from self-cultivation of the grains and potatoes group, which is typical of diets in rural areas. Also, although less than 30% of the farms cultivated dark green vegetables and other vitamin A-rich fruits and vegetables, among those women whose farms produced these foods, 75–85% reported their consumption. This suggests that greater consumption of vitamin A-rich foods from their own farms could occur among female farmers, if more grew these vegetables and fruits. Given that vitamin A deficiency is common in Ecuador, this could be an important approach in helping alleviate this nutrient deficiency. However, among the producers of the pulses, dairy, and eggs groups, less than half of the women on these farms reported consumption of these foods, which appear to be important sources of income from agriculture. Anecdotally, farm-fresh eggs have become popular in urban areas, which may limit their consumption by the farmers in favor of transport and sale in nearby cities. Future research should seek to understand how to best increase self-consumption of many of the nutritious foods produced on these farms.

Our results are consistent with other studies in the Americas that have shown only a small positive correlation between on-farm agricultural biodiversity and dietary diversity. In a recent study in Guatemala a positive correlation between agrobiodiversity and dietary diversity scores of children was observed; however, no relationship between agrobiodiversity and child anthropometric status was found [24]. Jones et al. [15] reported a positive association between farm-level agricultural diversity and DD scores of women in the Peruvian Andes, with a reported 1-unit increase in crop species richness associated with 17% higher odds of an increase in the MDD-W indicator. In an examination of 51 rural households in the Andes Highlands of Ecuador, Oyarzun et al. [16] identified a small positive correlation between the number of on-farm crop species and family-level dietary diversity. Several recent reviews of studies examining the relation between agricultural diversity and dietary diversity suggest that at best, the associations are quite modest. Jones [13] reported that of the 21 studies he reviewed, most reported weak positive associations between agricultural biodiversity and diet diversity. A larger review of 45 studies by Sibathu and Qaim [14] concluded agricultural biodiversity is positively associated with household-level and individual-level dietary diversity under some but not all circumstances. Only five of the studies reviewed had positive and significant associations for all sub-samples and indicators analyzed, 29 presented mixed results, and 11 studies found no significant positive associations.

Over 20% of the women in our study reported low dietary diversity, consuming less than five of the ten food groups. Among these women, starchy foods including rice, corn, potatoes, and bread were the common staples consumed, and only 10% reported consuming dairy products, only 24% reported ingestion of meat, fish, or poultry, and only 15% reported consumption of eggs. Given the low intake of these animal-derived foods, the average intakes of protein, zinc, and iron were significantly lower for the LDD compared to HDD women. In the regression analyses, we did not find the number of on-farm animals raised to be a significant predictor of DD despite the fact that 3 out of the 10 food groups (dairy, meat/fish/poultry, and eggs) used to determine DD scores are animal-derived foods. Possibly the low intake of such foods in the LDD group results from animals being raised primarily to generate income rather than providing food for home consumption. This phenomenon has been reported by Oyarzun et al. [16] in the Chimborazo Province of Ecuador. Note, however, that even among HDD women, intakes of animal foods rich in protein, iron, and zinc were low, with 60% reporting no consumption of dairy or eggs, and 40% reporting no consumption of meat, fish, or poultry. If the habitual diet does not include animal products, a wide variety of plant foods is considered important to ensure adequate intakes of protein, iron, and zinc. Thus as Jones [13] has recommended, diversification of crops targeting common nutritional deficiencies in a given geographical area may be more beneficial for public health than a sole focus on crop species counts.

The nutrition transition is characterized by increasing rates of obesity and comorbidities with simultaneous micronutrient deficiencies. Indeed, a secondary finding in the present study was that 67% of the women were classified as overweight or obese based on BMI values. In line with the double burden of over- and under-nutrition, among the 20% of the women who exhibited low dietary diversity with risk for inadequate nutrient intake, 65% were classified as overweight or obese, yet 33% answered affirmatively to at least four of the eight questions used to identify the magnitude of household food insecurity (data not shown).

This study has several limitations that should be noted. The observational design precludes determining causality and thus we can only speak to associations. The population studied was limited to women in the Imbabura province of Ecuador, where non-agricultural entrepreneurship and small business commerce is common, even in rural areas. Extrapolating to other populations should be done cautiously as socioeconomic level, off-farm income, and agricultural biodiversity vary considerably between geographic regions in Ecuador. Examining dietary and anthropometric characteristics of children residing on the farms would have been an excellent addition to the study, but we lacked adequate resources to include them. We queried the women as to the total land area of their agricultural farm plots, but unfortunately, inconsistencies in the measures reported suggest these data were not sufficiently accurate for use in this study. Quantifying the total number of animals raised for food is a crude measure given that the amount of food varies greatly between cows, pigs, and guinea pigs. The MDD-W is a useful method for summarizing population-level nutritional diversity for women of reproductive age [10,11]. It does not consider the total number of servings of each food consumed in the 24-h period. Nevertheless, despite its limitations and our sample including women of age beyond their reproductive years, we chose to use the MDD-W given its simplicity of use. Our survey was not able to examine all the factors related to agricultural diversity as it is highly complex. Other factors that can influence DD include market access and orientation, farming methods, women’s empowerment, off-farm income, and seasonality [10,11,13,14]. Nevertheless, the MDD-W has been shown to reflect risk for micronutrient deficiencies [11], hence its use in this study.

Despite efforts taken by the dietary interviewers to accurately measure types and quantities of food and beverages consumed by the study participants for the 24-h recall period, the average quantity of food consumed resulted in low calculated intakes of calories, macronutrients, and micronutrients, especially in those with LDD. However, food insecurity was common in this sample, more so in the LDD compared to HDD women, and during a given 24-h period, reduced food availability could readily result in low food intake. This is not to say that such low intakes are representative of habitual daily intakes that could be sustained long term. However, to exclude those with low energy intakes during the 24-h period in question would potentially mask the significance of food insecurity among these small farm holders. Nevertheless, the nutrient intake values derived from the 24-h recalls should not be used at face value, but rather we believe would be best viewed as providing support for the associations between lower food intake, less dietary diversity, lower financial resources and greater food insecurity. The LDD compared to the HDD group was older with lower body weight, likely resulting in somewhat lower energy intake requirements, which may have at least partially contributed to the energy intake differences. However, this would not account for the low reported energy intake in both groups. Finally, we recognize that economic resources play an influential role in determining agricultural productivity and nutritional status and acknowledge that our measure of per capita family income is a crude indicator of economic status, given the availability of non-monetary resources including land and livestock ownership. Nevertheless, per capita family income as estimated from respondents reported income from all sources, including agricultural sales, wages earned, non-agricultural sales (e.g., handicrafts), and government assistance, indicate that poverty is highly prevalent in this population. Seasonality (planting versus harvesting) can also affect available resources, but in this area of Ecuador, typically with three cycles of overlapping planting and harvesting per year, economic status does not wax and wane as much as in those areas with specific agricultural seasons.

Notwithstanding the limitations discussed above, there are several strengths of the study. It appears to be the first investigation in Ecuador to examine DD and agricultural diversity of rural, small holder farms where agriculture does not appear to be the primary driver of the regional economy and a wide variety of commercially processed foods and beverages rich in sugar, fat, and salt are readily available. We were able to survey a large sample of women from the region, which we believe to be representative of female small holder farmers in this Province. Highly trained agronomy and nutrition professionals were used as research assistants to collect data. They were local to the area and understood the common agricultural practices and possessed key cultural and nutrition-related knowledge unique to the area. Several of the research assistants spoke Kichwa in addition to Spanish and helped minimize any communication challenges.

In summary, we examined dietary diversity and agricultural food production diversity in a sample of female small holder farm managers in an area of Ecuador that has rapidly changed in recent years. The Imbabura Province is characterized by greater contribution to the economy of small business commerce, greater access of rural residents to urban food markets and evidence of continued nutrition transition from ancestral dietary patterns to greater consumption of commercially available food. Dietary diversity was higher among women residing on farms cultivating greater numbers of different food crops compared to women on farms cultivating fewer food crops, with this finding supported by the regression analysis demonstrating a significant independent association of dietary diversity among the women with total food crops produced on their farms. However, this association was modest, with other factors also independently related to dietary diversity including education level, family income, the magnitude of household food insecurity, and total energy intake. Overweight and obesity were common among women independent of their dietary diversity, suggesting that both over- and under nutrition (double burden) are prevalent in rural areas of this region.

Unfortunately, poverty and health inequities are all too common in rural areas of Ecuador, and greater emphasis must be placed on alleviating the sizeable burden shouldered by these residents. Our results suggest that local and national agricultural initiatives to enhance agricultural diversity on the small holder farms in the Imbabura Province could have some impact on the dietary diversity and nutritional status of their female caretakers. However, with the complex and rapidly changing global food systems and shifts in dietary patterns so well described by Popkin [25], other approaches to enhance intake of a variety of nutritious foods while simultaneously minimizing risk for overweight and obesity must also be encouraged. Public health policy could incorporate culturally appropriate educational programs specific to the region, draw upon the rich history of Indigenous peoples in Imbabura, and emphasize the best of traditional dietary patterns and farm cultivation of highly nutritious crops and animals for self-consumption to help alleviate the common deficiencies of protein, vitamin A, zinc, and iron. These programs could simultaneously help rural residents to develop knowledge and skills necessary to purchase (or trade for) healthy nutrient dense foods (including nutrient-enriched and fortified foods) from among commercially available items, thus capturing the best aspects of the traditional and transitional dietary patterns. Recognition of the rich cultural heritage of this region—yet currently one with high rates of both obesity and undernutrition—along with the opportunity for strong voices from Indigenous leaders, especially women, could encourage promotion of a comprehensive and inclusive vision for alleviating the health inequities seen in this population.

## Figures and Tables

**Table 1:** Descriptive characteristics of all study participants and the same participants divided by those with low dietary diversity (LDD < 5 food groups) compared to women whose diets met the minimum dietary diversity (HDD ≥ 5 food groups).

	All Participants (*n* = 558)	Lower Dietary Diversity (LDD) Score < 5 (*n* = 114)	Higher Dietary Diversity (HDD) Score ≥ 5 (*n* = 438)	*p*-Value for Difference
Variable	Mean ± SD	Mean ± SD	Mean ± SD	
Age (y)	44.4 ± 16.3	51.0 ± 17.7	42.6 ± 15.6	0.001
Formal Education (y)	5.8 ± 4.9	3.7 ± 4.3	6.3 ± 4.8	0.001
Height (m)	1.50 ± 0.1	1.49 ± 0.07	1.50 ± 0.06	NS
Weight (kg)	60.7 ± 12.0	59.2 ± 9.6	61.3 ± 10.4	0.04
BMI (kg/m^2^)	27.1 ± 4.3	26.6 ± 3.9	27.2 ± 4.3	NS
Energy intake (kcal/24 h)	1173 ± 470	959 ± 347	1229 ± 482	0.001
Protein intake (g/24 h)	39.4 ± 20.2	27.7 ± 14.7	42.4 ± 20.3	0.001
Iron intake (mg/24 h)	7.4 ± 4.2	5.4 ± 2.5	8.0 ± 4.3	0.001
Zinc intake (mg/24 h)	5.7 ± 3.8	3.6 ± 2.2	6.2 ± 3.9	0.001
Vitamin A (RE: µg/24 h)	335 ± 290	141 ± 172	385 ± 294	0.001
Dietary Diversity Score	5.7 ± 1.6	3.4 ± 0.9	6.3 ± 1.1	0.001

Abbreviations: BMI: Body mass index; RE: retinol equivalents; NS: not statistically significant (*p* > 0.05). *n* = 6 missing values for comparison of LDD and HDD.

**Table 2:** Multiple regression analysis identifying the change in the variance of dietary diversity scores of women from small holder family farms in the Imbabura Province as variables are added in a stepwise fashion going from model 1 (1 variable) model 5 (5 significant predictor variables).

Model	R	R Square	Adjusted R Square	SEE	R Square Change	F Change	Sig. F Change
1	0.402 ^a^	0.162	0.160	1.426	0.162	105.154	0.000
2	0.453 ^b^	0.206	0.203	1.390	0.044	30.019	0.000
3	0.484 ^c^	0.234	0.230	1.366	0.028	20.096	0.000
4	0.507 ^d^	0.257	0.251	1.347	0.023	16.564	0.000
5	0.513 ^e^	0.264	0.257	1.342	0.007	5.082	0.025

^a^ Model 1: Predictors: (Constant), Energy Intake [kcal]. ^b^ Model 2: Predictors: (Constant), Energy Intake [kcal], Household Food Insecurity. ^c^ Model 3: Predictors: (Constant), Energy Intake [kcal], Household Food Insecurity, Years of Education. ^d^ Model 4: Predictors: (Constant), Energy intake [kcal, Household Food Insecurity, Years of Education, Number of Different Food Crops Cultivated. ^e^ Model 5: Predictors: (Constant), Energy intake [kcal], Household Food Insecurity, Years of Education, Number of Different Food Crops Cultivated, Per Capita Family Monetary Income.

**Table 3:** Multiple regression analyses identifying the contributions of each of the 5 variables in Model 5 that contribute significantly to explaining the variance in the dietary diversity scores of the women from small holder farms.

Model 5	Unstandardized B	Coefficients Std Error	Standardized Coefficients Beta	*t*	Sig.
(Constant)	3.9	0.199		19.71	0.001
Energy (kcal)	0.001	0.000	0.306	7.8	0.001
Household Food Insecurity	−0.081	0.021	−0.146	−3.79	0.001
Years of Education	0.054	0.012	0.170	4.38	0.001
# Different Food Crops Cultivated	0.047	0.011	0.159	4.16	0.001
Per Capita Income	0.002	0.001	0.086	2.25	0.025
